# Experiences of Patients With Chronic Obstructive Pulmonary Disease Using the Apple Watch Series 6 Versus the Traditional Finger Pulse Oximeter for Home SpO2 Self-Monitoring: Qualitative Study Part 2

**DOI:** 10.2196/41539

**Published:** 2023-11-02

**Authors:** Yuxin Liu, Antonia Arnaert, Daniel da Costa, Pia Sumbly, Zoumanan Debe, Sylvain Charbonneau

**Affiliations:** 1 Ingram School of Nursing McGill University Montreal, QC Canada; 2 Academic Affairs, Teaching and Research Directorate Montreal West Island Integrated University Health and Social Service Centre Montreal, QC Canada

**Keywords:** Apple Watch, chronic obstructive pulmonary disease, pulse oximeter, qualitative descriptive, self-monitoring, smartwatch

## Abstract

**Background:**

Amid the rise in mobile health, the Apple Watch now has the capability to measure peripheral blood oxygen saturation (SpO_2_). Although the company indicated that the Watch is not a medical device, evidence suggests that SpO_2_ measurements among patients with chronic obstructive pulmonary disease (COPD) are accurate in controlled settings. Yet, to our knowledge, the SpO_2_ function has not been validated for patients with COPD in naturalistic settings.

**Objective:**

This qualitative study explored the experiences of patients with COPD using the Apple Watch Series 6 versus a traditional finger pulse oximeter for home SpO_2_ self-monitoring.

**Methods:**

We conducted individual semistructured interviews with 8 female and 2 male participants with moderate to severe COPD, and transcripts were qualitatively analyzed. All received a watch to monitor their SpO_2_ for 5 months.

**Results:**

Due to respiratory distress, the watch was unable to collect reliable SpO_2_ measurements, as it requires the patient to remain in a stable position. However, despite the physical limitations and lack of reliable SpO_2_ values, participants expressed a preference toward the watch. Moreover, participants’ health needs and their unique accessibility experiences influenced which device was more appropriate for self-monitoring purposes. Overall, all shared the perceived importance of prioritizing their physical COPD symptoms over device selection to manage their disease.

**Conclusions:**

Differing results between participant preferences and smartwatch limitations warrant further investigation into the reliability and accuracy of the SpO_2_ function of the watch and the balance among self-management, medical judgment, and dependence on self-monitoring technology.

## Introduction

According to the World Health Organization, chronic obstructive pulmonary disease (COPD) is the third leading cause of death worldwide [1]. Unlike other types of lung diseases, COPD disproportionately afflicts older adults, contributing to a debilitating impact on their quality of life through decreased exercise performance and functional capacity, with the eventual requirement of supplemental oxygen therapy to prevent exacerbations requiring hospitalization [2,3]. Whether supplemental oxygen therapy is indicated or not, noninvasive pulse oximetry has become a critical component of self-management for those with respiratory conditions such as COPD, as they are known to experience chronic room air hypoxemia due to persistent airflow limitation and gas exchange abnormalities [4]. For these individuals, commercially available finger pulse oximeters have enabled out-of-hospital peripheral blood oxygen saturation (SpO_2_) and heart rate (HR) monitoring and better home oxygen therapy management [5,6]. Moreover, the accessibility and portability of these devices have contributed to their prevalence for self-monitoring in individuals with COPD [7]. This has led to improved detection of acute hypoxemia events and prevention of exacerbations before the manifestation of visual cues such as cyanosis, thus reducing hospitalizations, particularly when used on a daily basis [8,9].

Pulse oximeters themselves have evolved significantly over the past decade, becoming increasingly integrated within the consumer wearable and mobile health market in the form of smartwatches, such as the Apple Watch Series 6, which provides several additional benefits beyond SpO_2_ measurement [10]. With the advancement of optical sensors, accelerometers, and gyroscopes, as well as improvements to on-device hardware-accelerated artificial intelligence (AI) for analysis of raw biometric data, the Apple Watch Series 6 has become increasingly capable of compensating for limitations of traditional pulse oximeters such as low perfusion, skin pigmentation, movement artifacts, and systemic sclerosis [11]. Although strong correlations were observed for HR and SpO_2_ measurements between the watch and the finger pulse oximeter, these findings were obtained under controlled conditions, and data were gathered at a single time point [11]. While results are promising, clinical trials that continuously monitor the SpO_2_ values of patients with COPD using the Apple Watch in naturalistic settings are needed. Due to a lack of reliable clinical data and an understanding of the AI algorithms used for health monitoring [12], the medical community has reacted with reluctance and skepticism, and questions of accuracy and reliability have been raised [13].

Considering the fact that the US Food and Drug Administration (FDA) and Health Canada currently regulate medical devices similarly, whether “static,” such as hip implants, or “dynamically updating,” such as smartwatches, regulation has impeded emerging mobile health technologies, which have become increasingly sophisticated [10]. In 2018, Apple Inc. obtained the FDA’s groundbreaking clearance for the Apple Watch, which was considered a Class II medical device with fall detection and advanced heart monitoring capabilities, such as low HR alert, irregular heart rhythm detection, and personal electrocardiogram monitoring [14,15]. The Apple Heart Study showed strong initial evidence that the Apple Watch might be a viable diagnostic tool in subclinical atrial fibrillation among relatively healthy young individuals [16,17]. Despite these promising results, researchers at the Mayo Clinic, who conducted a retrospective study, indicated that abnormal pulse readings resulted in an increased number of medically unnecessary emergency room visits [18], and the authors recommended that the FDA and Apple Inc. should carefully consider the unintended consequences of using the watch for asymptomatic atrial fibrillation.

Unlike the heart monitoring capabilities, Apple Inc does not have FDA clearance for their newly designed SpO_2_ feature, which was introduced during the COVID-19 pandemic era [19]. Due to the lack of governmental approval, the Apple Watch SpO_2_ feature should only be used for wellness and fitness purposes. Despite the fine print, consumers are using the device and considering it a medical tool [13]; often with limited knowledge about pulse oximetry in general [19]. Regardless of the need for reliable studies to validate smart wearable technologies, including the Apple Watch, it is imperative to understand the users’ experiences of using the Apple Watch Series 6 versus the traditional finger pulse oximeter for home SpO_2_ self-monitoring. Although the cognitive behavioral process underlying acceptance of the Apple Watch was explored in our previous study (add self-reference), we further elucidate the perceived differences in experience of using traditional finger pulse oximeters as compared with the Apple Watch. Therefore, the research question governing this qualitative descriptive study was: “Following the use of the Apple Watch and traditional finger pulse oximeter, how do patients with COPD compare their experiences monitoring their SpO_2_ between both devices?”

## Methods

### Design, Sample, and Recruitment

This qualitative descriptive study represents a subsequent analysis of interview data that were collected within a larger mixed methods research project evaluating an integrated telehealth nursing system including traditional finger pulse oximeters and the Apple Watch Series 6, which features reflectance pulse oximetry at the wrist. Although the larger project started in June 2020, the integration of the Apple Watch for select participants occurred in June 2021, from which this study draws its data. The sociodemographic data were outlined in our previous study (add self-reference). All participants were previously familiar with the traditional finger pulse oximeter, as it is standard in the management of COPD; however, none had any significant previous experience with the Apple Watch or any other similar wrist-worn pulse oximeter. All participants owned electronic devices, either a single smartphone or a combination of smartphone and tablet or personal computer.

### Data Collection and Analysis

Individual semistructured interviews were carried out through Zoom (Zoom Video Communications) with our participants after having experienced the Apple Watch for 5 months. Transcripts from the aforementioned interviews underwent primary open coding, then categories and themes relevant to device comparison, specifically around SpO_2_ measurement, were isolated and subsequently analyzed qualitatively, as described in the previous study (add self-reference). Strategies to enhance the trustworthiness of the findings remain the same among studies [20].

### Ethics Approval

Ethics approval was obtained from the research ethics committee of the Centre intégré universitaire de santé et de services sociaux de l'Ouest-de-L'Ile-de-Montréal (SMH #19-11) on August 16, 2019. All participants signed the consent form before the start of the larger study, and written information was provided explaining the study purpose, participant involvement, the right to withdraw at any stage, and data confidentiality. In June 2021, the 10 participants who agreed to use the Apple Watch were shown how to use the wearable and were monitored by the telenurse using the telemonitoring platform as described in the previous study (add self-reference).

## Results

### Overview

For accurate SpO_2_ readings, the watch requires a person to remain at rest with a flat wrist and a steady arm for at least 15 seconds. However, respiratory distress among patients with COPD is accompanied by erratic movements from the use of accessory muscles, resulting in the inability to yield accurate SpO_2_ measurements. This gap in the SpO_2_ continuous monitoring is described in the first theme, “unveiled blind spots of the watch*.*” Added to this measurement limitation, participants shared a discrepancy between their own subjective breathing problems and the associated O_2_ saturation values monitored by the watch and the finger pulse oximeter. Although a decline in trust toward the watch was expected following the limitations, some participants reported confidence in the device, as illustrated in the second theme, “leniency toward the watch margin of error.” The third theme, “perceived interdevice reliability,” addressed participants’ preferences for the traditional finger pulse oximeter, which was at arms’ reach, and the watch, which is hands-free and collects all health data continuously and spontaneously. Nonetheless, regardless of participants’ preferred device, they highlighted that the importance of recognizing, understanding, and acting upon their own COPD symptoms transcends the reliability of any monitoring device, as described in the fourth theme, “prioritizing personal health judgments.” Finally, the fifth theme, “reliance on health care provider’s medical judgment,” defined participants needing the reassurance of a health care provider to interpret the numbers and, if needed, start their COPD action plan.

### Unveiled Blind Spots of the Watch

Performing daily activities, according to participants P6-P10, can be exhausting and can trigger shortness of breath (SOB). For example, P6 shared: “I’m in the shower, washing up, and I’m getting really out of breath.” Others (P7-P8) stressed that walking in the neighborhood, doing routine clean-up, or household tasks may cause respiratory distress. Moreover, the mandatory mask-wearing during the COVID-19 pandemic was challenging, as explained by P8: “I get in a panic, like in the store the other day, the first thing I wanted to do, was rip off that mask.” In those moments of distress, P7 said: “When I experience SOB, I want to take it [O_2_ saturation level].” Participant P8 needed a device that “give her immediate attention of what her body’s going through.” The Apple Watch requirements for accurate SpO_2_ readings, such as being at rest and keeping the wrist in a stable position, are paradoxical with the capabilities of patients with COPD when experiencing dyspnea. Participant P7 explained the experience as follows:

When I am catching my breath, I make movements. Therefore, the watch is incapable of taking my measurements. I have to wait until I am calm.

She continued:

You can try and hold onto the sofa arm, but you can’t help but move when you are breathing hard. It shows a message: “Is your watch positioned properly,” or something like it, just when I need it.

Participant P6 pointed out that the “watch doesn’t work” when in distress; however, by the time she calmed down and recovered from the exacerbation, she said: “my oxygen level had gone up because I am doing the breathing exercises, taking deep breaths.” P6 questioned the necessity of having a watch for continuous monitoring and added: “Is it worth it? I don’t know.” Participant P7 made an interesting remark in terms of the added value of the continuous monitoring; saying:

I don’t think there is any variation in my [SpO_2_] data. My measurements at rest are never out of the normal range.

These experiences unveiled a gap, or “blind spot” of the watch to monitor participants’ SpO_2_ values continuously. The inability to obtain readings when breathing heavily causes participants (P7-P10) to consider the finger pulse oximeter as a more reliable option for capturing their O_2_ saturation levels. For example, P7 called the Apple Watch’s blind spot a challenge, further adding that “it is a challenge for the watch because with the manual oximeter, it [blind spot in the device] was never a problem.” P8 confirmed this behavior, saying, “If I feel bad, say I’m out of breath, I’ll do it manually with the finger thing [oximeter] and then I just breathe, do my exercises.”

Whether in respiratory distress or not, P10 found “the oximeter easier to use, it is easier to get your readings.” She further explained:

It doesn’t always work with the watch. I have to do it over and repeatedly whereas with the [traditional] oximeter I just take it on my finger, and it usually works right away.

P8 was well aware of the Apple Watch instructions, saying, “The technology of the watch relies on its [wrist band] positioning in a certain way.” Participant P9, however, had to “take her saturation level six or seven times before getting it properly fixed on her wrist.” She said:

It kept saying, “unable to read, make sure your watch is secure, comfortable,” and I would move it and I would still get that [message], so I was annoyed by it.

Although P9 was hopeful for the benefits of continuous monitoring, she left being very disappointed. She stated: “The watch was supposed to be easier and it’s not.” Additionally, for participants P5 and P10, whose wrists did not fit the standard bands, the difficulty collecting spot-check or continuous data is labeled a “blind spot of the device.” For example, P10, who has congestive heart failure due to severe COPD, is edematous peripherally, and her wrists are so swollen that they don’t fit any of 2 standard sizes available when purchasing the watch. She said: “If it had a more adjustable band, I wouldn’t have to work so hard to get it to work.” The wrist band fit so tightly that pitting marks remained after she removed the watch. These blind spots of the Apple Watch wearable form a gap in continuous data collection.

### Leniency Toward Watch Margin of Error

#### Discrepancies in SpO_2_ Measurements

The majority of participants (P1, P2, P5, P6, P8, and P10) noticed a significant discrepancy between the O_2_ saturation values collected from their finger pulse oximetry and the watch. For instance, P5 stated:

It is a six-point difference, that is huge. If I am 88% maybe I should be looking to go to the hospital, but I am at 94 [with the finger pulse oximeter], I’m okay.

Similarly, P1’s experience also reflected a mismatch; he stated that “especially at the beginning of the project, the data were completely different […] at one time, the watch was at 96% and the [other] oximeter at 91%.” Another participant, P8, indicated that the results reflected such big discrepancies that if you compare the watch and the finger pulse oximeter collected data, “you would think I’m collecting measurements from two different people, like the recordings of my husband and my recordings.” Sporadic large discrepancies were experienced by P6, who stated that “at times I have experienced differences of over 10 points and other times none.” With regard to the HR, P2 confirmed that “the difference [between the finger pulse oximeter and the watch] is contained between 1 or 2 units, never more.” He continued:

The problem is mainly the saturation readings. Sometimes the watch shows 87%, whereas it should ideally show 90 to 95%. At the other times it gives 100%, which is not correct. Then I take the oximeter and my saturation is normal 94 or 95.

Beyond the discrepancy between the watch and the traditional finger pulse oximeters, participants (P2, P5-P7, and P9) also noted variations between the devices and their own feelings. For example, P7 described that “sometimes the watch shows 100%; however, I do not feel like 100%.” Similarly, P9 recalled an instance where the telenurse called, saying, “My nurse called me because [the watch] showed I think it was at 83%, and I didn’t understand it either because I felt okay.” P5 supported these experiences as she was “kind of surprised” of the differences and stated: “I felt well but my vital signs collected were critically low.” These inconsistencies led participant P2 to have mistrust in the SpO_2_ values monitored by the watch. He revealed:

If I was confident about the number on the watch, I would not need the oximeter. But at this moment, I am not. It’s not complicated, I have seen a discrepancy in the numbers on the watch, I cannot trust those numbers alone. I cannot rely on it.

#### Default Confidence in the Watch

Despite the inconsistencies between the devices and compared with their feelings, 6 participants (P1 and P4-P8) surprisingly expressed having confidence in the watch SpO_2_ measurements. This confidence was rooted in having a default confidence in the watch due to distrust toward the manual oximeter. For example, P6 who experienced occasional discrepancies, when asked whether she lost confidence in the watch, replied: “No, not really, I still have confidence in the watch.” Even participant P7, who experienced drastic differences between devices, stated:

It is difficult to know which one is saying the truth. I have less confidence in the manual oximeter because I am not sure the battery is still good.

Other participants (P1, P4-P5, and P8) supported this statement. Participant P5 said:

The watch is much more reliable. I don’t know if it’s because the oximetry is now a year old, maybe the battery is becoming weaker.

P8 continued:

When you change the battery [of the oximeter], you know the light is brighter and it gives a more accurate reading; whereas the technology of the watch relies on you positioning the watch

which appears more reliable than dependence on batteries. The tech-savvy male participant (P1) had the impression that “the oximeter is less precise compared to the watch because we put it from finger to finger, hand to hand.” Participant P4 summarized this leniency toward the Apple Watch as follows:

If I had a vote of confidence, it would be for the wristwatch, because it is connected to the pulse. Whereas for the oximeter, I could have dirty fingers, the battery could be weak. If the battery [of the watch] is well-charged, then it cannot make an error.

An additional leniency toward the watch was rooted in the individual’s interpretation of the numbers provided by the device. For example, P8 indicated: “Maybe I’m supposed to read it [discrepancy between both devices] that way, my range is between 92 and 96.” P8 also added that there is leniency toward the Apple Watch because: “I’m not thinking this is curing my COPD. I’m thinking this as you’re trying to find out the best way of controlling somebody’s numbers.” Overall, the findings of participants reflected higher forgiveness and confidence toward the Apple Watch’s SpO_2_ data.

### Perceived Interdevice Reliability

#### Traditional Oximeter at Arms’ Reach

Due to its ease of use and availability, some participants (P2-P5) labeled the pulse oximeter as a reliable device. Participant P4 said:

I always have it in arms’ reach. As soon as I need it, I take it and put my finger into it. It does not take more than 10 seconds and I receive a reading.

Although this participant previously explained that his confidence in the accuracy of the results leans toward the watch, he clarified that in a situation of emergency, he would still prioritize the oximeter. This behavior was confirmed by P3:

If I was in distress, I would put my [Ventolin] puffer and I would take my oxygen level on the oximeter on my finger, I would not use the watch.

Although P4 enjoyed using the watch, he emphasized not yet mastering its usage, saying,

I do not understand the watch well enough yet, the first thing I will get [in emergencies] is the [manual] oximeter.

Another female participant, P5, shared:

I’ve used it [pulse oximeter] when my husband was not feeling well. I found that his heart was racing [...]. When we called the 911, I could give them that information, that was good.

Following this incident, P5 realized that the watch is not rapidly shareable like the finger pulse oximetry, which made it more reliable than the watch. When asked about the reliability between the 2 devices used, P5 responded, “I will say the oximeter.”

#### Hands-Free Continuous SpO_2_ Monitoring

On the contrary, participants (P6 and P8-P9) labeled the watch as a reliable device due to its physical ease of access. For example, P6 stated: “I do not keep the manual oximeter next to me,” therefore, she relied on the watch even during times of respiratory distress. Participant P8, however, anticipated that at the moment of sudden breathlessness crisis, first responders would “not dig in her drawer to get the finger thing,” but could use the watch. She continued that wearing the watch led to spontaneity of measurements collection, which also increased her perceived reliability. In fact, she explained:

I’m aware of more things happening in my body [...] When I was at Costco the other day [experienced respiratory distress], I had my watch and was able to check this

my saturation levels

For the majority of participants (P1 and P5-P9) the continuous monitoring feature of the watch was seen as a characteristic of reliability. Participant P1, who is very tech-savvy, shared:

The oximeter takes it [vital sign measurements] whenever I decide to take them. With the watch, the information regarding my oxygen is continuous, and allows me to see the fluctuations.

The watch’s ability to collect continuous data was further compared to inpatient intensive care, as per P5, who stated: “It would do it automatically and it’s like laying in an intensive care bed.” Moreover, the hands-free experience of the watch’s continuous monitoring ensured that the frequency of data collection no longer depends on manual initiation. P6 explained:

I find it interesting to have data more often. When I would take it on the finger, I would get one or two data entries. With the watch I have more details.

Even the participants, such as P9, who trusted the manual oximeter, shared that with the continuous monitoring, she felt safer. She explained:

The watch is wonderful. But for me, for my COPD, the oximeter was fine, I felt just as reassured […] Except for the fact that it [watch] monitors saturation on its own. That makes me feel even safer. But I felt safe with the oximeter also.

Above that, the nighttime continuous monitoring of the watch had allowed 2 participants (P1 and P6) to discover the nocturnal desaturations that they have been living with. P6 expressed:

I had a lot of difficulty sleeping. I would wake up questioning why is that? Then after I looked at the data collected overnight, I realised that my oxygen level was low at night.

Similarly, P1 stated that with the use of the watch overnight, he noticed that “often during the night his oxygen level would go down below 90 and at times below 86,” which gave him a clarification as to why he experienced difficulties sleeping.

#### Double SpO_2_ Verification

Before being enrolled in the larger study, participant P9 did not have access to a device that measured her oxygen saturation levels. She shared:

I had no way of tracking my HR or my oxygen level before I got the oximeter, I will always keep the oximeter once the project is over. It gives me reassurance that I am doing better.

Interestingly, for participant P2, having access to the largest quantity of health data possible was more important than the type of device used to measure her SpO_2_ values, saying, “For sure I find the [manual] oximeter more accurate than the watch, but like I said, I take both measurements, that is what reassures me the most,” in fact, “using both the watch and oximeter is a double verification.” In the case of participant P3, for whom the watch is a “family affair,” supported the statement that the device does not matter; however, she was open to any device as long as it prevents hospitalizations, saying,

The reason I am open to try a new device is the potential benefits it brings to my health since I don’t want to return to the hospital nor return onto oxygen.

Regardless of the device used, P3’s husband added with laughter, “In a situation of distress, she would call for me and I would get up to go help her with it.”

### Prioritizing Personal Health Judgments

Having access to reliable SpO_2_ data was important for all participants with COPD; however, any device, either the finger pulse oximeter or the watch, are limited to providing numbers. Total reliance on numbers produced by the devices appeared to conflict with participants’ (P1-P3, P5-P6, P8, and P10) self-perceived role in the management of their chronic condition. This was well said by participant P1:

Basically, there is a limit to what a computerized device can tell you. Devices do not have a judgment, even if algorithms are getting stronger.

Furthermore, in situations where his respiratory symptoms were deteriorating, P1 explained:

It is not the watch that will tell me [that I am in respiratory distress], I will feel it myself. In fact, I will know that I am too out of breath or exhausted.

He continued:

My first reflex is not to go see my blood oxygen level, but rather to decrease my activity, and to focus on the pace of my breathing to help calm myself.

He further highlighted that his physical symptoms are signals to stop all triggering activities: “If I continue and I persist, I know I will have terrible secretions, I will get a runny nose and I will have an urge to urinate.” In the same vein, P6, who had practically no concerns regarding the inconsistencies of the SpO_2_ measurements on both devices as she knows “her body and symptoms,” expressed: “It is not only about the numbers on the device but is about how I feel.” She continued:

I do not get worried if there is a difference. I am so used to being in the 80% (O2 saturation level), so when I see 90%-100% on the watch I tell myself that it is impossible.

For a person living with a chronic illness, having a baseline understanding of one’s own health prevents them from feeling stressed by external false alarms. Furthermore, participant P10, who has lived with COPD since 2016, agreed that she has developed a knowledge base of her physical symptoms. She shared:

Depending on the weather and the barometer, you have good days and bad days […] So, you know, early on if your day is going to be a bad day or a good day, you know by the barometer, by the weather.

Hence, in case of an emergency, P10 has learned to prioritize her physical symptoms above all numbers available, stating:

Number one for me is “I sit down” when I’m feeling uncomfortable, I’m having a hard time breathing, I sit down, and I breathe, I’ve learned how to breathe. And then I take my oxygen.

She continued:

If I have the watch on or I have the oximeter with me. I don’t always have both […] I go with whatever [device] is in hand.

According to P2, “the watch gives her an idea of her saturation and HR but does improve her health.” To conclude, P8 said:

I have the disease. There’s nothing I can do about it, but I talk to my body, listen to my body.

In contrast, for the female participant P5, the SpO_2_ numbers on the monitoring device must match her physical symptoms and not the other way around. In the situation where she felt fine but the watch kept showing low O_2_ saturation, P5 chose to repeatedly retake her vital signs until “she thought it was an accurate reading.”

### Reliance on Health Care Provider’s Medical Judgment

Importantly, in addition to knowing their body and understanding their physical symptoms, participants (P3, P5, and P8) were also relying on the telenurse’s interpretation of the submitted clinical data. Despite the new advanced digital health technologies, such as the Apple Watch Series 6, participant P5 verbalized: “I don’t see technology ever being able to replace the personal care, either from the doctor or the nurse, that we need.” P8 acknowledged this statement and added: “Technology is all these things, it’s the helpline to get to the result,” yet “I am not a medical expert. I can tell you how I feel but I’m not going to tell when to take my action plan. I want to speak to you [telenurse] first to reassure me that it’s the time to do it [take the action plan].” Similarly, participant P3 emphasized that although she has an action plan in backup, she does not want to start it on her own, saying, “I prefer that the nurses give me the okay. It’s not just some cough syrup, in the action plan there are antibiotics and cortisone. I prefer that a professional tells me when to start.” These cases reflect the reliance on the health care professional’s medical knowledge, assessment, and judgment also contribute to the patient’s management of COPD.

## Discussion

### Overview

Despite the difficulties in obtaining accurate SpO_2_ results when experiencing SOB and discrepancies between the watch and the traditional finger pulse oximeter, some participants had confidence and preference for the Apple Watch, as the device provided continuous passive measurements and other health-related data and allowed for meaningful feedback from a clinician. Interestingly, during exacerbations, participants would not rely on either reading but instead defaulted to their experienced physical symptoms and used their own judgment and health care providers’ clinical guidance for decision-making. Consequently, some points warrant further discussion: (1) the inherent limitations of wrist-worn pulse oximeters; (2) the inherent benefits of the Apple Watch; (3) the level of reliance on the Apple Watch; and (4) the potential for future hardware and software improvements.

### Inherent Limitations of Wrist-Worn Pulse Oximeters

When considering the blind spots we unveiled about the Apple Watch, an important distinction between all existing wrist-worn pulse oximeters is that they use reflectance pulse oximetry to obtain measurement, as opposed to the transmissive mode used by traditional finger-worn pulse oximeters [21]. Whereas transmissive pulse oximetry requires a light source and photodiode on either side of the measurement site (as with a finger probe), reflectance pulse oximetry is accomplished with both elements placed on the same side, allowing for measurement at many more sites, including the feet, forehead, chest, and wrist, as with the Apple Watch [21]. Unfortunately, this creates unique challenges, such as increased sensitivity to pressure and ambient light interference, requiring careful placement on the measurement site to maintain adequate contact [21]. The former challenge was borne out in our findings, with many participants having trouble measuring their SpO_2_ when experiencing respiratory distress, which compromised their ability to maintain adequate contact between the probe and their wrist. For patients with COPD, who often experience instances of respiratory distress causing difficulty controlling arm movement, this limitation is particularly relevant, as the most important indication for pulse oximetry in this population is for detection of acute hypoxemia requiring intervention. Contrastingly, for devices using transmissive pulse oximetry, such as with the traditional finger probe, the opposing light source and photodiode remain aligned on either side of the measurement site, increasing reliability in instances of increased movement.

### Inherent Benefits of the Apple Watch

As previously mentioned, the Apple Watch exemplifies a unique benefit of noninvasive wrist-worn pulse oximetry in providing an opportunity to track SpO_2_ more consistently. This is particularly relevant overnight, where comorbid conditions such as obstructive sleep apnea and congestive heart failure may increase the risk of nocturnal hypoxemia. In this study, 2 out of 10 of our participants stated that the continuous monitoring allowed them to detect nocturnal desaturations. Hence, the Apple Watch has demonstrated the potential to facilitate the diagnosis of concurrent obstructive sleep apnea in patients with COPD (referred to as “overlap syndrome”) [22]. Although home nocturnal oximetry has been implemented for the diagnosis of such complications in patients with COPD, this often requires prolonged monitoring for the detection of apnea or hypopnea events with additional information about sleep duration and arousals that cannot be easily obtained from home studies, therefore often requiring laboratory-based polysomnography studies for a more accurate diagnosis [22]. The Apple Watch, in fact, has the added capacity to combine sleep data with nocturnal oximetry to meet this demand for more comprehensive data required to support a comorbid diagnosis of sleep apnea for these patients.

### Level of Reliance on the Apple Watch

Despite this inherent advantage of the traditional finger pulse oximeter during events of respiratory distress, participants valued the passive nature of the Apple Watch for continuous self-monitoring. When compared with traditional finger pulse oximeters, the added convenience of the Apple Watch has led to preference over traditional devices for HR measurement [17], a phenomenon that appears to have carried over to SpO_2_ measurement as well. Moreover, 6 out of 10 participants in this study indicated a tendency to trust the Apple Watch readings over the finger pulse oximeter due to the perceived objectivity of the device when manual initiation is not necessary. This represents the combined effect of automation complacency and automation bias, where monitoring and vigilance of the device decrease as suspicion of error decreases over time. Unfortunately, passive sensors fail to collect subjective data about a patient’s symptoms (eg, pain or SOB) and therefore only present an incomplete account of the patient’s health status. In addition to the inability of obtaining accurate and reliable measurements when patients are experiencing respiratory distress, this significantly limits the feasibility of using this device for diagnostic purposes in this patient population.

Unfortunately, with the iteration of the SpO_2_ application installed on our test devices, the Apple Watch was unable to compensate for inadequate placement during respiratory distress events, rendering the device unusable for this purpose in naturalistic settings. This was surprising, given the lack of such challenges in the previous comparison carried out by Pipek et al [11] in a controlled setting. The accuracy and reliability resulting from a sterile and clinical setting contradict the purpose of self-monitoring devices, which are intended for noncontrolled home use. This study reveals the importance of taking into consideration the unique functional limitations of community-dwelling patients with COPD. In fact, weather-induced respiratory status changes, peripheral edema due to heart failure in severe COPD, and respiratory distress were all barriers to the successful use of the Apple Watch for continuous self-monitoring of SpO_2_.

### Potential for Future Hardware and Software Improvements

To address these limitations, one of the greatest advantages of smart wearables such as the Apple Watch is the possibility of dedicated hardware-accelerated machine learning to compensate for the wearer’s unique characteristics, including stability of placement on the wrist [10]. This feature allows for the combination of multiple data sources, such as integration with gyroscopes and accelerometers on the same device, and data from additional devices, such as the paired smartphone, to increase the signal-to-noise ratio and improve predictions through further aggregation and refinement [10]. Additionally, the Apple Watch incorporates the benefits of advanced mobile computing, such as over-the-air software updates and seamless integration with smartphone apps, empowering the end user through intuitive data visualization and meaningful alerts when biometric data exceeds predetermined thresholds [10]. For an increasingly smartphone-proficient older adult population, with 54% smartphone ownership in Canada according to most recent market research [23], these benefits are more likely to make meaningful impacts on their clinical outcomes by building from existing personal technology use and increasing engagement, leading to more timely therapy adjustment and intervention [24]. It remains to be seen if these iterative improvements to AI algorithms and onboard processing efficiency can overcome the challenges inherent in wrist-worn reflectance pulse oximetry and whether this will affect perceived accuracy and reliability for both patients with COPD themselves and their health care providers.

### Study Strengths and Limitations

This paper reinforced the importance of further exploration into the balance between the use of monitoring devices, self-management, and medical judgment. As our society faces increasing reliance on health monitoring devices, it needs to be acknowledged that it can be a double-edged sword. Although there are concerns for overreliance on technology reflected in the literature, there are also positive patient role changes related to the increased use of digital technologies. For example, it allows our health care system to increase remote monitoring, patient empowerment, and better consistency of care [9,10]. Furthermore, this qualitative research project conducted in a patient’s natural setting revealed the importance of taking into consideration the unique limitations of community-dwelling patients with COPD in future research. An important limitation of this project is the sample size and population selection. This paper focused on the population with COPD, which does answer the literature gap, but is not completely accurate to the demographics in our community. Most patients with chronic illnesses have a more complex profile than a single diagnosis. In fact, many patients with COPD have comorbidities that are interrelated, and all contribute to their health status, which was not discussed in this paper. Following this project, more research is needed as questions remain unanswered, such as: “How should health care providers integrate SpO_2_-enabled smartwatches like the Apple Watch into their treatment of community-dwelling older adults with COPD? How can we support patients in their adoption of new technologies while managing expectations about benefits and limitations?”

### Conclusions

This study provides new insights on the experiences of patients with COPD using both the traditional finger pulse oximeter and the novel Apple Watch as vital signs monitoring devices. The results discussed physical limitations, the lack of reliable SpO_2_ values, and the unexpected preference for the watch. Furthermore, the results covered how the health needs and their unique accessibility experiences impacted trust toward a device. The findings also unveiled a discussion on the significance of a device’s accuracy in a sterile environment versus the patient’s perspective on reliability in their natural setting and on the importance of clinical judgment as new technologies emerge. Within the participants’ experiences, the findings show a contrast between preferences and perceived reliability.

## Abbreviations

AI: artificial intelligence
COPD: chronic obstructive pulmonary disease
FDA: Food and Drug Administration
HR: heart rate
SOB: shortness of breath
SpO_2_: peripheral blood oxygen saturation

## Data Availability

Data cannot be shared publicly as per the institutional review board–approved protocol and approved consent form. Our approved consent specifically states that participant data that are potentially identifiable will not be shared. Given the nature of our qualitative transcripts and coding sheets, although stripped of identifiers, such as names, they contain potentially identifiable and sensitive participant information in even the most aggregate form if shared with certain individuals who are familiar with the context. Some of the aggregated data may be available with approval from the Research Ethics Committee. For further questions regarding the approved protocol and consent, the office can be contacted at recherche.comtl@ssss.gouv.qc.ca.
